# Inverse design of mode-locked fiber laser by particle swarm optimization algorithm

**DOI:** 10.1038/s41598-021-92996-1

**Published:** 2021-06-30

**Authors:** Alexey Kokhanovskiy, Evgeny Kuprikov, Anastasia Bednyakova, Ivan Popkov, Sergey Smirnov, Sergey Turitsyn

**Affiliations:** 1 Novosibirsk State University, Novosibirsk, Russia 630090; 2 Institute of Computational Technologies SB RAS, Novosibirsk, Russia 630090; 3 Aston Institute of Photonic Technologies, Aston University, Birmingham, B4 7ET UK

**Keywords:** Fibre lasers, Nonlinear optics, Solitons, Ultrafast photonics

## Abstract

A wide variety of laser applications, that often require radiation with specific characteristics, and relative flexibility of laser configurations offer a prospect of designing systems with the parameters on demand. The inverse laser design problem is to find the system architecture that provides for the generation of the desired laser output. However, typically, such inverse problems for nonlinear systems are sensitive to the computation of the gradients of a target (fitness) function making direct back propagation approach challenging. We apply here particle swarm optimization algorithm that does not rely on the gradients of the fitness function to the design of a fiber 8-figure laser cavity. This technique allows us to determine the laser cavity architectures tailored to generating on demand pulses with duration in the range of 1.5–105 ps and spectral width in the interval 0.1–20.5 nm. The proposed design optimisation algorithm can be applied to a variety of laser applications, and, more generally, in a range of engineering systems with flexible adjustable configurations and the outputs on demand.

## Introduction

Despite the breakthrough achievements of the recent decades (see e.g.^1–9^ and references therein), design of mode-locked fiber lasers is still a challenging scientific and engineering problem. The main reason for this is the intrinsic nonlinearity of the elements forming fiber laser cavity and resulting non-trivial spatio-temporal field dynamics due to interplay between the key physical effects. In the mathematical terms, the operators describing action of each element in the resonator are not commuting, leading to complex nonlinear dynamics of light and dependence of the laser output radiation on the order of the system elements^10^. Wide range of the current and emerging applications, including micro machining, metrology, spectroscopy, microwave photonics, bio-medical applications, telecommunications and many others, stimulates active research on development of new advanced approaches for cavity design and control of pulsed regimes in fiber lasers. Underlying nonlinear physical effects and competing mechanisms of pulse formation create a great variety of possible lasing regimes. Mastering fiber laser nonlinearity paves the way to control this variability of the characteristics of the output radiation. To provide flexibility of the feedback based control, cavity parameters should also be easily variable and adjustable either electronically or optically.

Recently, new methods to generate optical patterns by electronic control of semiconductor optical amplifier or an electro-optical switch have been demonstrated^11,12^. However, these methods are limited to microsecond-scale pulse patterns. The control becomes more challenging in the case of ultra-short pulses, when interplay between chromatic dispersion and Kerr nonlinearity starts to play significant role in the spectral and temporal shaping of generated optical pulses. For instance, stable generation of the classical solitons strictly obeys the balance condition between chromatic dispersion and nonlinearity. As a consequence, soliton fiber laser generates pulses with temporal duration and spectral width varying in a limited range. The energy of the (fundamental) solitons generated in the mode-locked fiber lasers with anomalous dispersion is also limited by sub-nanojoule level due to the Kelly side-bands instability^2,4,13^. Relatively simple laser output pulse shapes, such as e.g. Gaussian or sech waveforms make easy their characterisation. However, there are more interesting possibilities in terms of shapes and properties of the output laser pulses in the lasers with nonlinear intra-cavity dynamics.

Dissipative soliton^1,2^ (DS) arguably is one of the most attractive types of the laser pulses for tailoring and manipulating spectral-temporal profile of the optical field. Numerical modeling indicates that a wide range of temporal and spectral pulse shapes can be obtained by tuning parameters of the propagation equation^1,14,15^, in a certain way related to the parameters of the laser cavity. In our previous work we have shown that distributed control of nonlinear phase accumulation inside the figure of eight (F-8) fiber laser cavity^16–18^ via gain of the amplifying media provides an efficient and practical way to adjust duration, energy and degree of the coherence of the generated pulses^19^. Peak power clamping effect associated with appearance of dissipative soliton resonance^20^ may be effectively avoided by insertion of an additional amplifying stage inside the laser cavity^21,22^.

It is worth noting, that general experimental realization of the F-8 laser allows only the pump powers to be tuned, leaving the other passive elements fixed. Optimization of the passive elements of the laser cavity, such as spectral filter bandwidth, coupling ratios and lengths of the passive and active fibers in the experiment is time-consuming and expensive. In this case, numerical modeling is a more practical approach to the optimization problem. However, knowledge of the cavity parameters is not always easily available.

There are two main approaches in optimisation. The first is the forward problem: given certain choice of laser architecture with variable parameters what are the possible lasing regimes? Though more straightforward, this approach requires substantial computational resources and it is not guaranteed that the desired target parameters can be achieved. The second approach is the inverse problem: given a desired laser output, how we can design the laser to generate it? In many aspects, the inverse problem is more challenging, because the solution space is typically non-convex, meaning there exist multiple local optima. In many systems gradient-based methods are not efficient for the inverse design purpose. Therefore, it is an important challenge in modern laser science to identify the efficient design techniques given a desired laser output and practical constraints.

Machine learning algorithms have been already shown to be highly effective in solving optimization problems arising in photonics^23–27^. Particular type of the optimization algorithm should be chosen taking into account specific features of the fitness function. In general, a function describing the area of stable pulse generation in the multi-dimensional space of cavity parameters is complex and often discontinuous. Stochastic algorithms that do not rely on the gradient of the fitness function, can be more efficient here than conventional gradient-based algorithms widely used in the artificial neural networks.

**Figure 1:**
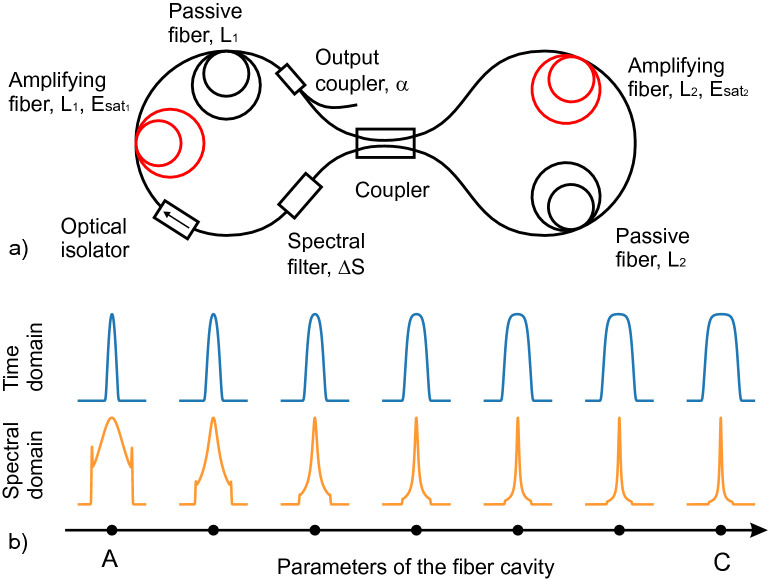
(**a**) Principle scheme of the F-8 fiber laser cavity. (**b**) Variation of the spectral-temporal properties of generated dissipative soliton during linear scalability of the cavity parameters.

Here we apply the particle swarm optimization (PSO) algorithm for the inverse system design^28–31^ in the F-8 laser cavity (Fig. 1). Taking the advantages of special architecture of the laser cavity and dissipative soliton features we examine PSO algorithm to design pulses with parameters on-demand. We combine the standard black-box PSO approach with the heuristic considerations based on the general dissipative soliton theory and knowledge of the physics effects underlying pulse evolution in the resonator.

## Figure-eight laser set-up and variability of the output dissipative solitons

To demonstrate our general approach we examine a particular laser set-up depicted in Fig. 1a. This scheme is chosen for illustrating the PSO-based approach, because it is relatively simple in experimental implementation, but possesses a rich and complex nonlinear dynamics of the laser radiation in cavity. Numerical modeling of the intra-cavity light evolution was performed using the master model that is a version the Ginsburg-Landau equation (GLE), and that is also known, as the generalized nonlinear Schrödinger equation (NLSE)^1,4,5^ (see “Methods” section). A NLSE-based numerical model was applied to F-8 fiber laser with two independently pumped amplifying stretches of fiber at both loops of the cavity (Fig. 1a).

The proposed scheme allows to control coefficients of the effective saturable absorber, that is not easily available in fiber laser schemes using the nonlinear polarisation evolution or carbon-nanotubes. As variable parameters we used $E_{{\mathrm {sat}}\,1}$ and $E_{{\mathrm {sat}}\,2}$ (saturated energies of the amplifying fibers at uni-directional loop and NALM correspondingly), $L_{1}$ and $L_{2}$ (the lengths of the passive fiber inside NALM and uni-directional loop correspondingly), and $\Delta S$ (bandwidth of 6th-order super-Gaussian spectral filter), $\alpha$ (ratio of the output coupler). Note that the variation of $E_{{\mathrm {sat}}\,1,2}$ of the amplifying fibers corresponds to variation the pumping power of the fibers in experiment.

We observe a high variability of the available output pulses that have clear characteristics of the dissipative solitons^1,2^. We have found that linear scaling of a vector comprised of the listed above cavity parameters leads to transformation of spectral-temporal distribution of generated output dissipative soliton as illustrated in Fig. 1b. It is seen that the output pulses are variable in both temporal width and spectral bandwidth. The used F-8 architecture allows to generate dissipative soliton with varying time-bandwidth product. Such pulses also have variable phase modulation (chirp characteristics) that makes possible their further temporal compression. Knowledge of the chirp characteristics of the output pulses also offers more rich possibility of the pulse manipulation, including nonlinear transformation of the shape and temporal-spectral characteristics. Note that pulse shapes shown in Fig. 1b are well-known in the dissipative soliton theory and can be approximated analytically. We will exploit below this fact to offer some simplified description of this variability of the output pulses. Modern laser systems with nonlinear intra-cavity dynamics extend characteristics of pulsed lasers beyond conventional Guassian or sech shapes to more complex waveforms.

Thus, the considered figure-eight fiber laser system is easily implementable with a flexible design and adjustable cavity elements, and also this scheme provides for a large variability of the output pulses, making the inverse design problem interesting and non-trivial. However, achieving exact parameters of the output pulses on demand requires robust method to determine parameters of the laser cavity. In the next section we demonstrate how to utilise Particle Swarm Optimization algorithm to implement mapping between the desired pulse characteristics and the laser system design parameters.

## Particle swarm optimization algorithm

The central part of our work is the application of the particle swarm optimization algorithm to the inverse design of the considered figure-eight laser system. The gradient-free PSO algorithm was implemented to map the desired properties of the output pulses with the parameters of the F-8 laser cavity. The algorithm starts with random initialization of N particles that have their positions and velocities in the swarm. In our case, position of a particle is a vector of the cavity parameters and velocity determines the change of this vector at the next step of the algorithm. For each particle we simulate pulse formation with NLSE-based model of F-8 fiber laser. Then, particles are valued by fitness function in order to estimate their proximity to target pulse. Velocities of the particle are found at each step using the following formula:

1 $$\begin{aligned} {\mathbf {v}}_i \leftarrow \omega {\mathbf {v}}_i + \phi _{p} {\mathbf {r}}_p\times ({\mathbf {p}}_i-{\mathbf {x}}_i)+\phi _{g}{\mathbf {r}}_g\times ({\mathbf {p}}_g-{\mathbf {x}}_i), \end{aligned}$$

where $x_{i}$—current set of parameters of i-th particle, $p_{i}$, $p_{g}$—previous best position of i-th particle and whole group’s previous best position, $r_{p}$, $r_{g}$—random values updating each iteration step within zero and one, $\phi _{p}$, $\phi _{g}$, $\omega$– hyperparameters affecting on convergence speed. Particular values of the hyperparameters are described in the “Method” section. Positions of the particles are updated and algorithm repeats until fitness function does not reach a target threshold value, or the change of the fitness function does not exceed corresponding variation threshold level.

The fitness function is designed to define the target (desired) output pulses. In this particular example, it was constructed with commonly used in practice parameters: full-width half maximum (FWHM) of the temporal and spectral distribution:

2 $$\begin{aligned} f(\Delta T, \Delta \lambda ) = \alpha _{1} \cdot \frac{|\Delta T - \Delta T_{target}|}{T_{max}} + \alpha _{2} \cdot \frac{|\Delta \lambda - \Delta \lambda _{target}|}{\lambda _{max}}, \end{aligned}$$

where $\Delta T$ and $\Delta \lambda$—FWHM of the temporal and spectral distributions of the current particle, $\Delta T_{target}$ and $\Delta \lambda _{target}$ are desired values, $\alpha _{1}$ and $\alpha _{2}$—temporal and spectral weights, $T_{max}$ amd $\lambda _{max}$—widths of temporal and spectral windows of the numerical model. Note that by introducing the specific form the fitness function and weights $\alpha _{1}$, $\alpha _{2}$ one can control the characteristics that are the most important for particular applications.

## Results and discussion

### Spectral-temporal characteristics of the output pulses

**Figure 2:**
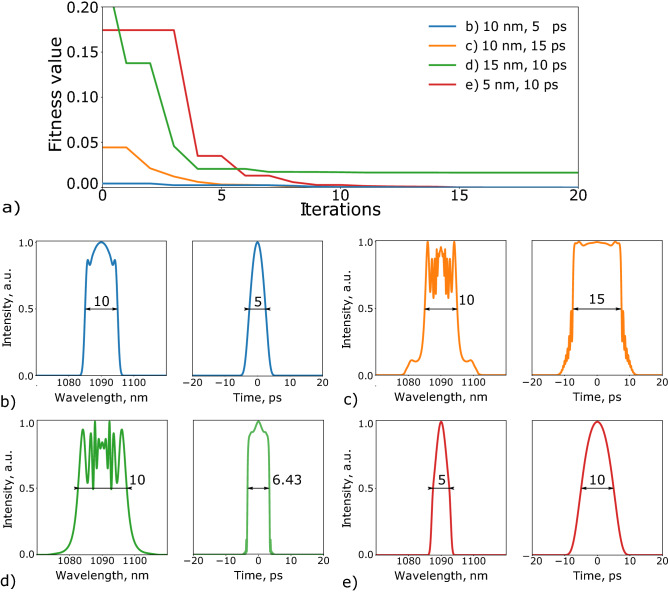
(**a**) Convergence of the fitness function for different target output pulse parameters. (**b**–**e**) Several examples of the spectral profile and temporal waveform of the dissipative solitons generated on demand.

The advantage of the inverse design approach is that we start from the definition of the target set of the output pulse parameters. To demonstrate the capabilities of the PSO algorithm we examined different sets of pulse parameters: spectral bandwidth in the interval from 0.1 to 20 nm and temporal duration in the interval from 1 to 100 ps. Some examples of a multitude of the obtained solutions are demonstrated in Fig. 2b–e.

Figure 2a shows that the algorithm requires just a relatively small number of iterations to reach the desirable output pulse parameters. However, a green line also illustrates limitation of the method. Indeed, in the most cases fitness function converged fast (examples are given by the Fig. 2b, c, e and, respectively by blue, orange and red lines in Fig. 2a except for the pulses with spectral bandwidth exceeding 15 nm). In such a case fitness function value clamped to relatively high level and remained the same for large number of iterations, as shown by the green line in Fig. 2a. Figure 2d demonstrates a solution found with the algorithm, while the required parameters were $\Delta T_{target}$ = 10 ps and $\Delta \lambda _{target}$ = 15 nm. Restarting the algorithm and tuning its hyper-parameters did not solve the problem. Case under the letter d) illustrates a simple fact that this system has limitations in terms of what can be produced on demand. In other words, PSO allows to explore a space of possible outputs on demand, but only within the limits imposed by the given laser system.

**Figure 3:**
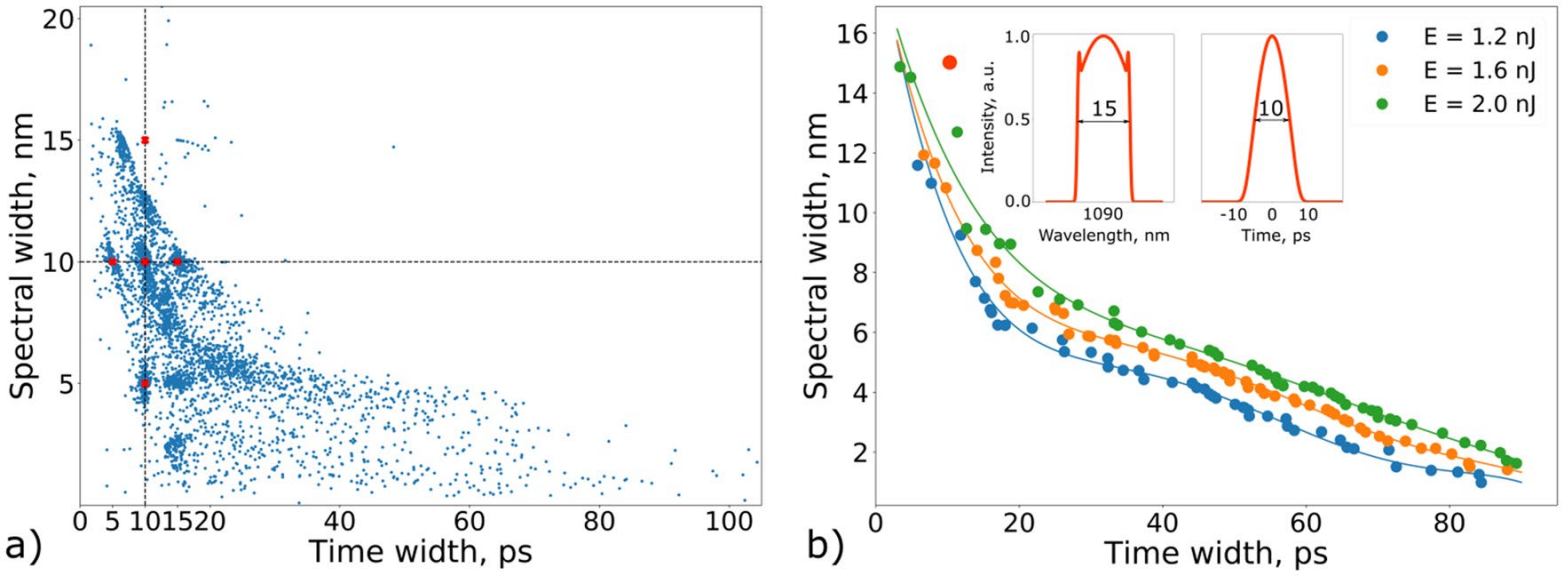
(**a**) Visualization of all obtained stable regimes obtained during the PSO algorithm execution. Each blue dot on the graph corresponds to a certain laser output with the given bandwidth and pulse width. Red crosses denote the target parameters that were set to the PSO algorithm. (**b**) Colored dots correspond to the boundaries (in the space of parameters) of the regimes, the energy of which does not exceed 1.2 nJ (blue dots), 1.6 nJ (orange dots) and 2.0 nJ (green dots). Lines have been obtained as the approximation by polynomials of the 5th degree for each set of these points. Inset shows spectrum and temporal profile corresponding the the red circle (bandwidth of 15 nm and pulse width of 10 ps).

The inverse design procedure has been used to explore all the intermediate solutions. A huge database of the laser cavity parameters was collected together with corresponding parameters of the generated pulses. Summary of the solutions (shown in the plane (spectral bandwidth, pulse width) is demonstrated in Fig. 3a. Each point on this graph corresponds to a certain laser output with the given bandwidth and pulse width. Depending on the application, one can choose operational regime that will lead to these pulse parameters. Note that though a great variability of the available operational regimes have been studied in the fiber lasers with the nonlinear polarisation evolution (NPE), see, e.g.^32^, the NPE-based fiber lasers have limited reproducibility of the generated pulses. This can be mitigated by the use of the self-tuning and machine learning-based control (see, e.g.^19,33–37^), however, this requires additional processing and control systems. Note that the right upper part of the graph, which corresponds to pulses with relatively large temporal duration and spectral width, is empty. This clearly indicates that time-bandwidth product (and likely, the pulse energy) of the generated pulses is restricted by the selected ranges of variable cavity parameters.

Next, we fixed several energy levels (1.2, 1.6 and 2.0 nJ) and chose the pulses with such energies, having the largest time-bandwidth product (Fig. 3b). For pulses with energy 1.2 nJ the maximum value of the time-bandwidth product was 189.8 ps$\cdot$nm. Selecting pulses with energies around 1.6 nJ we found that the maximum value of time-bandwidth product increased to the value 228.3 ps$\cdot$nm. Hence, reaching the higher value of the time-bandwidth product requires increasing the energy of the generating pulses. Maximum energy of the generated pulses is mostly governed by the gain of the amplifying fibers of the laser cavity. We increased upper limit of saturation energies of the amplifying fibers in order to increase overall gain of the laser system. Indeed extending the ranges of the saturation energies up to 1 nJ allows to find laser cavity parameters to generate 5 ps pulses with 15 nm bandwidth (see inset in Fig. 3b).

It should be noticed, that, evidently, the energy of the generated pulses cannot increase infinitely due to onset of stimulated Raman scattering (SRS). One of the options to mitigate SRS effect is to increase the diameter of the fiber that will increase the energy threshold value of the effect. Also performing near the threshold value, NLSE model has to be modified to take into account the SRS effect^38^.

### Semi-analytical approximation of the output pulses

We observed that variability of the output waveforms can be to some extent approximated using a single analytical expression—a complex function *U*(*t*):

3 $$\begin{aligned} U(t)= & {} \frac{A}{\sqrt{\cosh (\frac{t}{\tau }) + B}} \cdot \exp \left[ -i\frac{\beta }{2}\ln \left( \cosh (\frac{t}{\tau }\right) + B)\right] , \nonumber \\ E= & {} \int {|U(t)|^2 dt}= A^2\; \tau \; \int {\frac{ds}{\cosh (s)+B}}=A^2\;\tau \;Q(B). \end{aligned}$$

here *A*, *B*, $\beta$, $\tau$,—are real constants, corresponding to pulse characteristics. Energy *E* of such pulses is a function of the parameters $A, \tau$ and *B* : $E=A^2\; \tau \; Q(B)$, where $Q(B)= \int {\frac{ds}{\cosh (s)+B}}$. Note that this waveform appears as a solution of the complex Ginsburg–Landau equation with the quintic term and was already used for description of dissipative laser solitons^14,39^. However, we would like to stress that we do not make here any link between our system and the traditional Ginzburg–Landau equation with the quintic term, because the latter model is applicable only for cavities with uniformly distributed parameters, when variation of the pulse parameters is insignificant along the cavity^1,4^. We rather approximate and map various complex regimes into the analytical expression Eq. (3) that allows to mimic the observed pulse properties.

The function (3) is a handy tool to illustrate a variability and flexibility of spectral-temporal properties of the dissipative solitons. For instance, variation of the output waveforms from point A to point C in Fig. 1b resembles pulse shape transformation with increasing parameter B. By changing the scaling parameter B one can transfer between two qualitatively different types of a dissipative solitons. The first type ($|B|<1$) is characterized by broad spectrum and narrow temporal distribution, while the second type ($|B|>1$) has narrow spectral spike and relatively wide temporal distribution. Note also that the function (3) also gives description of the root-mean-square (RMS) characteristics such as pulse width $T_{RMS}$ and spectral width $\Omega _{RMS}$.

4 $$\begin{aligned} T_{RMS}^2 = \frac{\int { t^2 |U|^2 dt}}{\int {|U|^2dt}} = \tau ^2 \times \frac{R_{1}(B)}{Q(B)}, \;\;\;\;\; \Omega _{RMS}^2= \frac{\int { |U_t|^2 dt}}{\int {|U|^2 dt}}=\frac{1}{4\,\tau ^2} \times \frac{R_{2}(B)+ \beta ^2 R_{3}(B)}{Q(B)} \end{aligned}$$

Here $R_1(B)=\int {\frac{s^2 ds}{\cosh (s) +B}}$, $R_2(B)=\int {\frac{\sinh ^2(s) ds}{[\cosh (s) +B]^3}}$, and $R_3(B)=\int {\frac{ds}{[\cosh (s) +B]^3}}$ This, in particularly, means that dependence of the RMS spectral width on the temporal RMS pulse width can be presented as follows:

5 $$\begin{aligned} \Omega _{RMS} = \frac{\sqrt{Q_1(B) +\beta ^2 Q_2(B)}}{T_{RMS}}, \;\;\;\; Q_1(B)=\frac{ R_{1}(B) \times R_{2}(B)}{4\,Q^2(B) },\;\;\; Q_2(B)=\frac{R_{1}(B) \times R_{3}(B)}{4\,Q^2(B)} \end{aligned}$$

**Figure 4:**
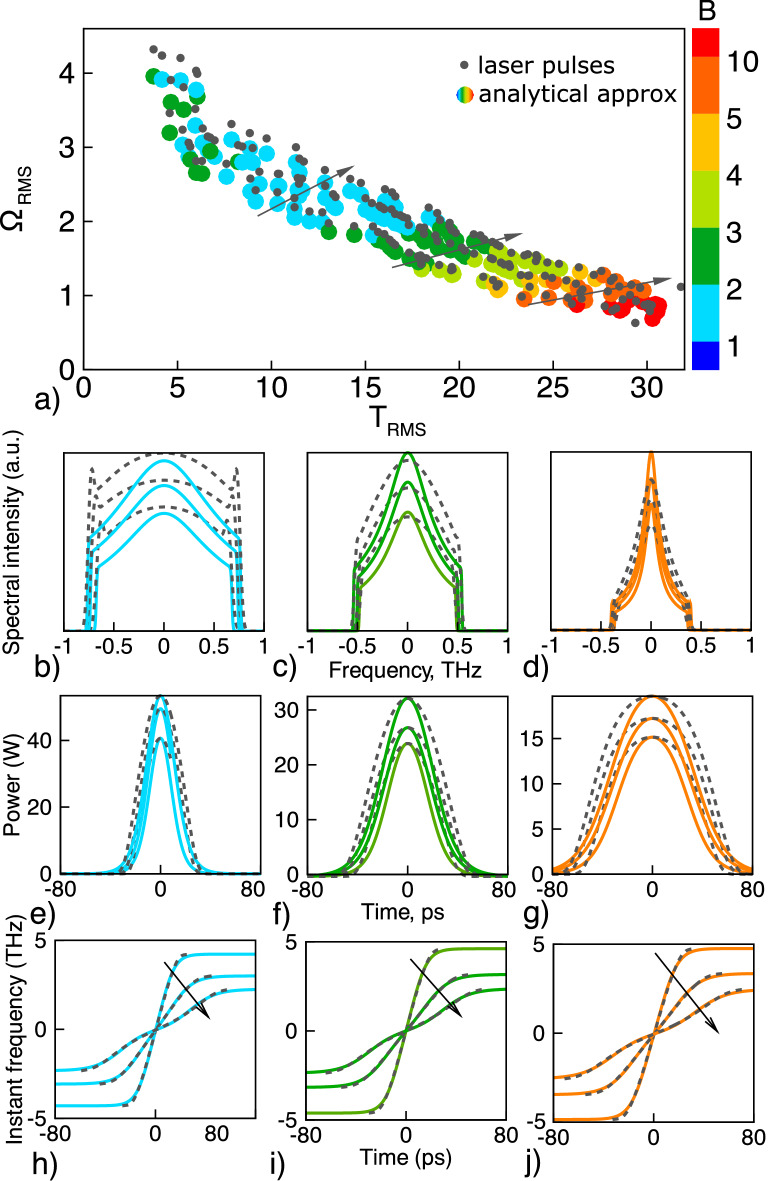
(**a**) RMS temporal and spectral width of the laser pulses and their analytical approximation (3). Color shows the value of parameter B. (**b**–**j**) Comparison of the pulse shapes of the laser pulses (dashed lines) and their analytical approximations.

We apply the complex function *U*(*t*) defined by Eq. (3) to approximate the observed shapes of the output laser pulses, depicted by dots in Fig. 3b. We found the unknown parameters $\tau$, *B*, $\beta$ and *A* of the analytical expression and compared it with the laser pulses (the optimization algorithm is described in the “Methods” section). Figure 4a demonstrates that analytical function describes variety of the laser pulse shapes.

It reproduces well RMS characteristics, while temporal and spectral shapes of the pulses are slightly different. The case is that after amplifier a pulse propagates in the long passive fiber with normal dispersion and then leaves the cavity. During this propagation its shape becomes more parabolic and can’t be approximated by inverse hyperbolic cosine.

The found value of the parameter *B* allows us to distinguish between different types of the pulses—pulses with wide spectra characterized by steep edges and flat-topped wide pulses with narrow spectra. As the parameter B gradually varies along the energy lines, all intermediate pulse shapes are also attainable. Another interesting observation is a conservation of *B* during transition between the energy levels, which allows us to find self-similar pulses with different energies. The arrows in Fig. 4a show directions of this transition, while the corresponding pulse shapes are shown by lines of the same color in Fig. 4b–g. These observations allow us to assert that the PSO algorithm can be applied to inverse laser design. In other words, we have shown, that any relation between spectral and temporal pulse duration is attainable (within some boundaries imposed by physical limitations such as Raman scattering).

The instantaneous frequency behavior, considered as the main fitting criteria for mapping analytical function and simulated laser pulse (detailed description is given in the “Methods” section), is shown in Fig. 4h–j. Pulse chirp is positive as the DS formation occurs in the cavity with normal dispersion, but there are some distinct features between the pulses, corresponding to different values of B parameter. The instant frequency is almost linear if B close to unity (Fig. 4h), which indicates the possibility of high-quality pulse compression. If the value of B parameter increases (Fig. 4j), the instant frequency remains linear only near the top of the pulse. Shape of the instant frequency manifests itself in the shape of a pulse spectrum with a high narrow peak.

### Discussion

The machine learning is already transforming the way in which photonic systems are designed and optimized. Fiber laser with its inherent complex spatio-temporal nonlinear dynamics with corresponding high-dimensional phase space is an interesting and practically important test-bed for application of the machine learning based design methods. Here we apply gradient-free PSO method to inverse design problem of figure-8 fiber laser. We demonstrated that a great variability of the pulses generated in such system can be used for offering pulses on demand with pulse duration in the range of 1.5–105 ps and spectral width in the interval 0.1–20.5 nm. These pulse parameters are consistent with those obtained by various scientific groups using both numerical and experimental studies of mode-locked fiber lasers, see e.g^14,20,40^. In the works^20,41^, using a similar NLSE equation and saturated amplification model, experimental and numerical results are in good agreement. Therefore, there should be no issue with future transfer of the developed algorithm to the experimental systems. We would like to stress the difference between these works and ours: we applied PSO algorithm to optimize properties of dissipative soliton instead of comprehensive grid search of the proper laser parameters. We also observed that in a range of parameters the multitude of the output pulses can be well approximated by the relatively simple analytical expression for the dissipative chirped solitons. The deviations of the output pulses and the analytical expression is the most pronounced in the temporal domain where the analytical expression cannot fully reproduce parabolic-type temporal waveform of the laser pulses. The developed design optimization PSO algorithm can be applied to a variety of laser applications, and, more generally in the photonic systems with adjustable configurations.

## Methods

### Numerical model

The intra-cavity light dynamics has been modelled by the conventional generalized nonlinear Schrödinger equation (NLSE):

6 $$\begin{aligned} \frac{\partial A^{\pm }}{\partial z}= i\gamma |A^{\pm }|^2A^{\pm }-\frac{i}{2}\beta _2\frac{\partial ^2 A^{\pm }}{\partial t^2}+\frac{g_0/2}{1+E/E_{\mathrm {sat}}}A^{\pm }, \end{aligned}$$

where $A^{\pm }(z,t)$ is the optical field envelope with upper index ± corresponding to the forward and backward counter-propagating waves inside the bi-directional loop, *z* is the longitudinal coordinate along the fiber, *t* stands for time in the retarded frame of reference, $\gamma$ and $\beta _2$ are non-linear and dispersion coefficients, respectively, $g_0$ and $E_{\mathrm {sat}}$ denote unsaturated gain coefficient and saturation energy of the amplifying fiber, $E = \int {(|A^{+}|^2+|A^{-}|^2)\,{\mathrm {d}}t}$ is the total energy of the counter-propagating waves. In order to simulate pulse propagation through a passive fiber, we use the same Eq. (6) with $g_0=0$, whereas for pulse propagation inside the unidirectional cavity loop, we take $A^{+}\equiv A(z,t)$, $A^{-}\equiv 0$.

Equation (6) was integrated numerically using the well-known step-split Fourier method^42^. The time window considered in the model was equal to 300 ps. The time grid included 4096 points, which corresponded to 0.0125 nm spectral resolution. More details about numerical integration of Eq. (6) in the case of bi-directional loop can be found in Ref.^21^.

### Particle swarm optimization algorithm

We empirically find optimal values of hyper parameters of the equation (1). We have used a swarm with N = 50 particles, $\phi _{p} = 2$ , $\phi _{g}$ = 2.1, $\omega$ = 0.4. These values provided sufficient converging speed of the fitness function and avoided clamping to its local extremum value. The algorithm requires 10 iteration steps to find desired stable solutions in average corresponding to 500 simulation of the laser cavity.

Six cavity parameters were used as the particle parameters: saturation energies $E_{sat1}$ and $E_{sat2}$, passive fiber lengths $L_1$ and $L_2$, output coupler ratio $\alpha$ and spectral filter width $\Delta S$. These parameters varied within predetermined ranges. The saturation energies were in a range from 0.05 to 0.1 nJ, the passive fiber lengths were limited in a range from 0.5 to 100 m, the value of the filter width varied between 1 and 15 nm, and the output coupler ratio was in a range from 5 to 50%. Fiber nonlinearity $\gamma$ and chromatic dispersion $\beta _2$ were the same for both active and passive fibers and were equal $\gamma = 4.66 \frac{1}{\text {W km}}$, $\beta _2 = 24.3 \frac{ps^2}{\text {km}}$.

Simulations were performed on computational cluster of Novosibirsk State University (12-core Intel Xeon E5-2680v3 2500 MHz). Average converging time for stable solution was 29.8 min.

To speed up massive calculations we also add conditions breaking simulations: (1) The field energy is less than a certain value (2) The pulsed regime was not stabilized after 1000 passes of the laser cavity. (3) Pulsed regime did not stabilize after 60 min of calculation. These conditions filter slow-converging and unstable solutions.

### Analytic function approximation

The values of parameters $\tau$, *B*, $\beta$ and *A* of the function *U*(*t*) (3) were found using step-by-step solution to the optimization problem. On the first stage the value $\tau$ was found with a fitting function equal to RMS temporal width difference between laser pulse and *U*(*t*). On the second step the value of parameter *B* was obtained. We considered a shape of instantaneous frequency (phase derivative) as a fitting criteria. Then several iterations were made on $\tau$ and *B* as they both affect pulse duration. On the third stage $\beta$ was found by minimizing difference between the maximum instantaneous frequency of *U*(*t*) and the laser pulse. After this step the spectra of both pulses include the same frequency range. And on the last fourth stage the value of parameter *A* was adjusted so that the amplitudes of the pulses become equal.
